# A Rapid Assessment of Disaster Preparedness Needs and Resources during the COVID-19 Pandemic

**DOI:** 10.3390/ijerph18020425

**Published:** 2021-01-07

**Authors:** Lawrence A. Palinkas, Benjamin F. Springgate, Olivia K. Sugarman, Jill Hancock, Ashley Wennerstrom, Catherine Haywood, Diana Meyers, Arthur Johnson, Mara Polk, Carter L. Pesson, Jessica E. Seay, Caroline N. Stallard, Kenneth B. Wells

**Affiliations:** 1 Suzanne Dworak-Peck School of Social Work, University of Southern California, Los Angeles, CA 90089-0411, USA; 2 LSU Health Sciences Center—New Orleans, School of Medicine and School of Public Health, New Orleans, LA 70112, USA; bspri2@lsuhsc.edu (B.F.S.); okacsi@lsuhsc.edu (O.K.S.); awenne@lsuhsc.edu (A.W.); cpesso@lsuhsc.edu (C.L.P.); jseay@lsuhsc.edu (J.E.S.); cstall@lsuhsc.edu (C.N.S.); 3 Pennington Biomedical Research Center, Baton Rouge, LA 70808, USA; jillhancock@gmail.com; 4 Louisiana Community Health Outreach Network, New Orleans, LA 70119, USA; gray.catherine1949@gmail.com; 5 St. Anna’s Episcopal Church, New Orleans, LA 70116, USA; diana@stannanola.org; 6 Lower Ninth Ward Center for Sustainable Engagement and Development, New Orleans, LA 70117, USA; ajohnson@sustainthenine.org; 7 National Alliance on Metal Illness-New Orleans, New Orleans, LA 70115, USA; fireflyseaclans@yahoo.com; 8 Center for Health Services and Society, Jane and Terry Semel Institute, Department of Psychiatry and Biobehavioral Sciences, David Geffen School of Medicine, Department of Health Policy and Management, Fielding School of Public Health at UCLA, Los Angeles, CA 90095, USA; kwells@mednet.ucla.edu

**Keywords:** COVID-19, disaster preparedness, disaster response, natural disasters, community-based organizations

## Abstract

*Background:* This year has seen the emergence of two major crises, a significant increase in the frequency and severity of hurricanes and the COVID-19 pandemic. However, little is known as to how each of these two events have impacted the other. A rapid qualitative assessment was conducted to determine the impact of the pandemic on preparedness and response to natural disasters and the impact of past experiences with natural disasters in responding to the pandemic. *Methods:* Semi-structured interviews were conducted with 26 representatives of 24 different community-based programs in southern Louisiana. Data were analyzed using procedures embedded in the Rapid Assessment Procedure-Informed Community Ethnography methodology, using techniques of immersion and crystallization and focused thematic analysis. *Results:* The pandemic has impacted the form and function of disaster preparedness, making it harder to plan for evacuations in the event of a hurricane. Specific concerns included being able to see people in person, providing food and other resources to residents who shelter in place, finding volunteers to assist in food distribution and other forms of disaster response, competing for funds to support disaster-related activities, developing new support infrastructures, and focusing on equity in disaster preparedness. However, several strengths based on disaster preparedness experience and capabilities were identified, including providing a framework for how to respond and adapt to COVID and integration of COVID response with their normal disaster preparedness activities. *Conclusions:* Although prior experience has enabled community-based organizations to respond to the pandemic, the pandemic is also creating new challenges to preparing for and responding to natural disasters.

## 1. Introduction

The year 2020 saw the confluence of two major crises influencing the health and well-being of people living in the United States and elsewhere. The first, which has received the most attention, has been the COVID-19 pandemic. Since its appearance in Wuhan, China in late 2019, the pandemic has resulted in 85 million people who have tested positive for the coronavirus and over 1.8 million deaths worldwide [1]. In the United States, there were 20.5 million confirmed cases and 350,775 deaths attributed to the coronavirus as of 4 January 2021. The pandemic has led to widespread disruption of social and economic life as nations have struggled to contain the spread of the coronavirus through preventive efforts such as social distancing, closure of certain sectors of the economy, and restriction of public gatherings. Unemployment rates rose dramatically during the first few months of the pandemic to levels not seen since the Great Depression of the 1930s [2] and have yet to return to pre-pandemic levels as nations experience subsequent waves of infections [3].

The second crisis has been a marked increase in the frequency and severity of disasters due to acute weather events such as hurricanes. With a total of 30 named tropical storms, 2020 now holds the record for the most named storms, the most active hurricane season on record. The previous record was 28 storms in 2005. One of these storms (Iota) developed into a Category 5 hurricane, four storms (Laura, Teddy, Delta, and Eta) developed into Category 4 hurricanes, and eight other storms developed into Category 1–3 hurricanes [4]. Since May 16 (earlier than the usual start of the season, which is June 1), these events have resulted in over $40 billion in damages and 362 deaths [5].

Nowhere in the United States has this confluence been more evident than in the Gulf of Mexico region in general and the state of Louisiana in particular. As of 6 January 2021, there have been 326,648 individuals who have tested positive for the coronavirus and 7635 COVID-related deaths [1]. Five storms made landfalls in Louisiana, the most on record in one season [5].

Along with other forms of natural disasters and acts of terrorism, infectious disease outbreaks or pandemics often result in a surge in demand for health care and social services. Recent analyses show that in addition to the delivery of intensive care for individuals who have become severely ill due to the virus, the need for services in the general population has also increased [6,7,8,9]. For instance, the need for mental health services has increased as the prevalence of mental health problems has risen as a result of economic losses and unemployment [8,10], fear of infection [7,9,11], and isolation and confinement [7], including among incarcerated populations [12]. Health care systems have been overwhelmed by spikes in the number of patients needing hospitalization and treatment of COVID-related conditions, while experiencing a decline in revenues due to a reduction in services for other health problems [13,14]. However, there have been no studies to date that have examined the impact of the pandemic on disaster preparedness and recovery.

Similarly, there have been concerns that the hurricane season could potentially exacerbate the effects of the pandemic in vulnerable regions of the U.S. [15,16]. Efforts to protect the general population from hurricane hazards, including large-group evacuation and sheltering, could potentially necessitate the easing of efforts to slow the pandemic through social distancing and sheltering in place [17]. On the other hand, the efforts of community-based organizations (CBOs) to support individual and community-level resilience to reduce the impact of natural disasters in regions affected by climate change may also help to address similar COVID-related impacts such as morbidity and mortality, separation from loved ones, loss of employment, disrupted social networks and supports, services redistribution, and hazard exposure [18,19,20,21,22]. It is therefore unclear whether a priority given to dealing with one crisis limits the ability to respond to the other crisis, and whether the experience of responding to acute weather events such as Hurricane Katrina has helped or hindered efforts to respond to the pandemic.

This paper summarizes the information collected from 26 stakeholders participating in a rapid assessment of community priorities, strengths, and needs as a result of the COVID-19 pandemic. Our aim was to answer two specific questions: (1) How has the COVID-19 pandemic impacted disaster preparedness and recovery in Louisiana, a state that has been especially vulnerable to natural disasters such as hurricanes and floods; and (2) How has the state’s past experience with disasters impacted its response to the pandemic?

## 2. Methods

### 2.1. Setting

The Community Resilience Learning Collaborative and Research Network (C-LEARN) (NCT03977844) is a community partnered research trial that aims to determine best practices and intervention approaches to build and support disaster-prone communities in Louisiana [23]. The trial was developed based on experience in services implementation and research in disaster response in Louisiana, particularly with a focus on mental health and community engagement, as well as on work in addressing mental health and disaster preparedness and response in Los Angeles [24,25,26,27,28,29,30,31]. C-LEARN was designed to be conducted in two phases. In Phase 1, key informant interviews with community stakeholders throughout southeast Louisiana were conducted to identify emergent themes in community strengths, weaknesses, and priority areas as related to mental health and disaster. Results from Phase 1 informed activities in Phase 2. Phase 2 is a two-tiered, randomized trial of two interventions at each tier. In Tier 1, participating agencies, providers, and administrators were randomized to either (a) technical assistance (TA) in support for finances, housing, and disaster response, or (b) Community Engagement and Planning (CEP) for multi-sector coalition support + TA. In Tier 2: clients of agencies that participated in Tier 1 were randomized to receive one of two text message-based interventions: (a) a Community Resources (CR) guide and (b) the CR guide and a text message-administered Cognitive Behavioral Therapy-based mental health intervention (CR + eCBT) [23,24].

The C-LEARN study completed Phase 1 [32,33], agency recruitment for Phase 2, and intervention training and implementation. Participant recruitment was initiated, but the onset of the COVID-19 pandemic interrupted individual participant recruitment shortly after being initiated. This substantially shifted health care, community agency, and community members’ needs and priorities. Following the primary aim of C-LEARN to build and support disaster-prone communities in Louisiana, with input from participating communities and study investigators, the primary goal and design of this study was redirected to conduct a rapid qualitative assessment of community priorities, strengths, and needs as a result of the COVID-19 pandemic and its intersection with potential concerns about climate events such as hurricanes that were pending as the pandemic was occurring. The academic and community stakeholders had participated post-Katrina in applying the Rapid Assessment Procedure (RAP) to inform future intervention efforts [25], and selected the expanded version, Rapid Assessment Procedure-Informed Community Ethnography (RAPICE) for the new study framework [34].

Consistent with the principles and practice of Community Partnered Participatory Research (CPPR) [26], both the C-LEARN project and the framing of the RAPICE phase [34] were performed through a community–academic partnered approach with extensive involvement of community stakeholders in New Orleans, Baton Rouge, and two predominately rural parishes (St. Bernard and St. John the Baptist) in southeastern Louisiana. A description of stakeholder involvement in participant recruitment, data collection, and data analysis is provided below.

### 2.2. Participants

Eligible participants for individual interviews were required to be English speaking, aged 18 years old or older, and identified as a community leader or employed by a CBO. Participant recruitment began with individuals who previously completed interviews in C-LEARN Part 1. Additional participants were identified and contacted potentially eligible community leaders not previously affiliated with C-LEARN through snowball sampling using contact lists provided by Part 1 interviewees and/or C-LEARN Leadership Council members. Snowball sampling was selected as a purposive sampling strategy for use in this study because the high level of network linkages among disaster-related CBOs previously identified in the study region [23] would ensure a representative sample for qualitative research [35]. Potential participants were then contacted via email using the LSU Health Sciences Center—New Orleans IRB-approved participant invitation letter, or by phone using the invitation letter text as a script. Participants were provided with another key information sheet, both by email and verbally before beginning the interview.

### 2.3. Data Collection

Using a semi-structured interview guide co-developed by members of the C-LEARN Leadership Council, all interviews were conducted over the phone or online using the Zoom platform. An invitation to participate was sent to interviewees via a LSUHSC-NO email with the verbal consent form attached. Upon acceptance, study staff read through the consent form with them again at the time of the interview. Participants provided information on their own demographic characteristics and the characteristics of the clients served by their agencies. They also provided information on their agency’s mission and how that mission and associated activities had been affected by the COVID-19 pandemic. Specific questions related to disaster preparedness included the nature of services provided; impact of the pandemic on services delivery; partnerships with other agencies and organizations; and trainings participated in with respect to disaster preparedness and recovery, mental health services, and other services. Interviews lasted between 45 min and one hour and were recorded and transcribed for analysis. Individually identifying information was removed at the time of transcription.

### 2.4. Data Analysis

Analysis of the data obtained from the semi-structured interviews followed a protocol embedded in a procedure for conducting rapid assessments known as Rapid Assessment Procedures-Informed Community Ethnography (RAPICE), an adaptation of Rapid Assessment Procedures-Informed Clinical Ethnography [34]. In this instance, rather than conduct research in settings where trained clinicians were involved in collecting ethnographic data, we involved community leaders who were members of the C-LEARN Leadership Council to participate in the data analysis components of the RAPICE methodology. The method involves rapid evaluation of key themes, including as “ethnography”, direct interaction of a trained qualitative methodologist with interviewees or interviewers and community stakeholders to inform/clarify key themes and enrich descriptions. Interviews were conducted between June and July 2020. Analysis was completed in August and September of 2020. All study activities were reviewed and approved by the Louisiana State University Health Sciences Center—New Orleans Institutional Review Board, with relying approvals from the University of Southern California and University of California, Los Angeles Institutional Review Boards.

Specifically, the first author reviewed transcripts of the semi-structured interviews and performed a preliminary analysis using the immersions/crystallization [36] and focused thematic analysis techniques [37] that are part of the RAPICE methodology. These preliminary findings were then presented to the C-LEARN Leadership Council, academic collaborators, and to the interviewers to gain more insight into the data and its context and to obtain a preliminary interpretation of its meaning and significance. Over 650 double-spaced pages of interview transcripts and memos, along with notes from the meetings with the C-LEARN Leadership Council, were then coded by the research team to condense the data into analyzable units. Segments of text ranging from a phrase to several paragraphs were assigned codes based on a priori (e.g., from a semi-structured interview guide) or emergent themes (also known as open coding). Following the open coding, codes were assigned to describe connections between and within categories (also known as axial coding). Based on these codes, QSR NVivo 12 and Atlas.ti were used to generate a series of themes arranged in a treelike structure connecting text segments grouped into separate categories of codes or “nodes.” Consistent with previously explicated RAPICE methods [34], a discussion then ensued until both the research team and Leadership Council reached consensus as to the meaning and significance of the data. Inter-rater reliability in the assignment of specific codes to specific transcript segments was assessed for five randomly selected transcripts. For all coded text statements, the coders agreed on the codes 84% of the time, indicating good reliability in qualitative research [38].

## 3. Results

A comparison of the population size, median household income and percent of population living in a FEMA designated flood zone in the four parishes served by the CBOs represented by study participants is provided in Table 1. The average age of participants was 48.5 (range = 28 to 70) years. The majority (61.5%) were non-Hispanic white; 30.8% were Black; one participant (3.8%) was Latinx and one participant (3.8%) was Vietnamese. Three-fourths of the participants lived in Orleans Parish, and most represented local-level organizations. Participants represented 24 community-based agencies and organizations that provide a wide variety of services including environmental and social justice issues impacting underserved communities, community health promotion, health and mental health services, disaster preparedness and recovery, funding of community initiatives, community development, faith-based services, affordable housing, child welfare advocacy and support, and criminal justice reform. A little less than half of the agencies (42.3%) represented by study participants served all ages. Some agencies represented specific age groups such as older adults (23.1%) and youth (11.5%). While one-third of the agencies served clients from all socioeconomic strata, two-thirds served clients representing low- or low–middle-income clients. The majority of agencies (61.5%) represented operated in majority Black communities. Information on languages spoken by clients and health insurance status was less reliable as it was not routinely collected by agencies. Similarly, estimates of the proportion of clients served who were at risk for COVID-19 were not verified, although two-thirds of participants believed that 50% or more of the clients served by their agencies were at risk.

### 3.1. Services Provided

Of the 24 agencies represented by study participants, six provided services to a single group of clients, such as pre- and post-natal care for pregnant and parenting women, primary care services for individuals with opioid use disorder, leadership development for incarcerated adults, advocacy and services for LGBT older adults, advocacy for victims of child maltreatment, and patient navigation for Spanish-speaking clients. Other CBOs provided specific services to low- and middle-income residents, including case management for social services such as food assistance or access to government benefits, (*n* = 3); housing assistance (*n* = 3), HIV prevention (*n* = 2), community wellness and health promotion (*n* = 3), workforce development (*n* = 2), and interpersonal violence harm reduction (*n* = 2). Five CBOs provided direct mental health services; four were primarily engaged in neighborhood beautification, wetlands restoration, and environmental green projects, and two provided funding for community health and education initiatives. One CBO provided faith-based services, including food assistance, homeless outreach, and youth development programs.

Nineteen of 24 agencies provided specific disaster preparedness and recovery services. Two additional agencies provided ad hoc services such as emergency financial assistance or deliveries of food and water to survivors when called upon to do so. Seventeen participants reported prior experiences with disasters in the region since Katrina. A list of services provided pre- and post-disaster and illustrations of how they are provided is included in Table 2. Pre-disaster services provided included community education and webinars, training of volunteers and first responders in disaster preparedness and response training, evacuation support (resident registration, development of evacuation plans), assisting other CBOs in preparation and implementation of disaster preparedness plans, distribution of emergency supplies, fundraising and financial support, and environmental risk management. Post-disaster services provided included material assistance to disaster survivors (food, water, supplies, financial), post-disaster debris removal, delivery of mental health service, rescue and recovery of survivors, emergency health care support, fundraising, and follow up with evacuees.

### 3.2. Impact of the COVID-19 Pandemic on Disaster Preparedness and Recovery

Representatives of three agencies reported that the pandemic had little or no impact on the services that they provided. Of the remaining agencies, a number of challenges were reported that were attributed to the pandemic (Table 3). For instance, eight agency representatives reported concerns with providing routine response and recovery services virtually and not face-to-face in the event of a natural disaster. Six participants expressed concerns about how to evacuate people given the provisions for social distancing and concerns that available locations for evacuation were currently experiencing surges in the number of positive cases, hospitalizations and deaths. As a result of these concerns, several of the clients and community members served by these organizations planned to shelter in place and not heed mandatory evacuation orders in the event of a hurricane (*n* = 3). Four participants reported having difficulty recruiting and training volunteers to help with disaster response and recovery. Three participants noted that households were experiencing difficulty gathering food and supplies in anticipation of the hurricane season due to unemployment and loss of income resulting from the pandemic, while three participants also reported concerns about providing food and other supplies during a disaster. Three participants stated the pandemic has forced their organizations to place disaster planning activities on hold. Two other participants stated that they were experiencing difficulty soliciting funds from businesses they traditionally relied upon for support due to massive layoffs of employees and declining revenues. One participant also reported that non-profit organizations had shifted their priorities to funding COVID-related activities. One participant reported difficulty preparing communities to plan for natural disasters because of the focus on COVID-19, and another participant reported that the pandemic led her agency to focus on racial/ethnic equity in disaster preparedness.

The focus on the pandemic has also produced a sense of “disaster fatigue” in the study region. One of the C-LEARN Community Leadership Council members noted that New Orleans had two near misses and one direct hit (Zeta) by hurricanes this season and that the combination of social unrest and the pandemic has contributed to feelings of anxiety and emotional exhaustion, making residents feel psychologically ill equipped for respond to a hurricane if one were to strike.

### 3.3. Impact of Disaster Preparedness and Recovery on Responding to the COVID-19 Pandemic

Participants were also asked how their experiences with past disasters have enabled CBOs to respond to the COVID-19 pandemic. Three participants indicated that nothing had prepared them for the pandemic because the pandemic is really different from a hurricane. As one participant noted: “We can’t see people face-to-face like we did in hurricanes” (Participant 5). Another participant commented that “controlling the response to a mix of a health and economic disaster is a lot harder to predict the long-term arc of so we usually say in a disaster, there’s short-term, mid-term, long-term and then mitigation” (Participant 7). Still another noted that unlike a hurricane, the pandemic “doesn’t have an end point, and you can’t escape it” (Participant 26).

“And so, I saw a lot of people go into hurricane mode and hurricane mode is about waiting to see what happens and cleaning up afterwards…. And pandemic mode is about prevention. And so that was the kind of mental leap that was pretty tough for people that I saw”.

(Participant 22)

Another participant reported that while agencies possess an infrastructure and have plans in place to address environmental disasters, they have virtually none to respond to the pandemic.

However, reliance on previous experience with hurricanes and floods as a lens for responding to the COVID-19 pandemic is more nuanced as many participants identified both similarities and differences between the current pandemic and previous natural disasters. This is reflected in the following statement offered by one program director:

“I definitely think that’s true. I have certainly heard my clients, especially in the beginning, liken this current moment or liken COVID to their experience with Hurricane Katrina more specifically as it relates to like the uncertainty of things, the fear kind of associated with the unknown. So, in my counseling sessions, the fact that some clients were able to make that connection did I think facilitate a type of resiliency around feeling like they were going to get through this. In other words, it facilitated a way of coping, I think. And then, at the same time, yeah, I would say where it stopped was that COVID doesn’t have an end point. Well, two things. Let me back up. Well, no, I stay where I am. It doesn’t have an end point, and you can’t escape it. So, whereas with Hurricane Katrina, you could leave the Gulf Coast region and go somewhere else and get reprieve. With COVID, you can’t go anywhere and escape it. Everyone is affected. So, I think that has created a unique condition unlike Katrina. Where is the reprieve, where is the safety? There is none. So, I think that layer of this experience as a unique sort of ecosystemic crisis brought about... Again, on the one hand, there was a space of resiliency and being able to liken it to Katrina. But then this idea of it’s very much so not like Katrina. Therefore, it creates another level of stress and frustration and worry and anxiety and challenge”.

(Participant 26)

Several participants identified a number of resources acquired through their experience of preparing for and responding to past disasters that equipped them to cope with the pandemic (Table 4). The greatest resource of all of the agencies and organizations represented in this study was their partnerships with other community-based agencies and organizations. Participants’ CBOs engaged diverse partner agencies in disaster preparedness, which included Ready.gov, Metropolitan Human Service Authority, NOLA Ready, Voluntary Associations Active in Disasters (VOAD), and the Office of Homeland Security and Emergency Response. Participants also mentioned partnerships with agencies and churches that provided assistance in rebuilding homes damaged by natural disasters and financial assistance to disaster survivors.

Another resource used by the community-based agencies and organizations during the pandemic has been access to training webinars that are specific to the pandemic. Agencies also used pre-COVID training experiences to address pandemic-related issues such as mental health. Six participants reported applying pre-COVID training through the C-LEARN collaborative in their responses to their pandemic; another six participants reported receiving COVID-relevant disaster preparedness training from other sources.

Delivery of mental health services to clients and community members who had experienced natural disasters since Katrina is another resource that community-based organizations engaged in disaster preparedness and response have contributed during the pandemic. Prior experience with natural disasters also enabled certain agencies to develop a reputation as a source of information for a lot of people, and to earn trust of the community. Two participants mentioned that their role in disaster preparedness and response also provided them with an ability to respond to the pandemic faster than CBOs in other parts of the country with little experience in natural disasters. One participant mentioned that this role also provided them with a healthy donor base needed to begin delivering services such as food and mask distribution.

In addition to resources, the experience of preparing for and/or responding to natural disasters provided several agencies with important lessons that were being applied in responding to the pandemic. The most important lesson learned from prior experiences with natural disasters has been the ability to bring people together for a common purpose. This was cited by six participants. Past experiences with natural disasters also provided an increased confidence in the ability to respond to the pandemic, to coordinate activities with other agencies and communities, and to connect pandemic survivors with necessary resources including food and financial assistance. Other lessons learned from past disasters included the importance of keeping on top of disseminating information, assessing community needs, avoiding mission creep (i.e., taking on responsibilities that exceed agency capability or mission scope), upgrading technology, taking care of one’s own mental health, and staying connected to clients. Finally, one participant noted that communities and community residents especially vulnerable to natural disasters and their impacts were also those especially vulnerable to the impacts of the pandemic, suggesting that prior experience in helping these communities to respond to hurricanes and floods taught them how to respond to the pandemic in an under-resourced community.

## 4. Discussion

The aim of this study was to determine how the COVID-19 pandemic impacted disaster preparedness and recovery in Louisiana and how the state’s past experience with disasters has impacted its response to the pandemic. The results suggested that the pandemic has imposed constraints on other forms of disaster preparedness and response, in particular in relation to the context for this study, climate events such as hurricanes. The respondents appeared to be evenly divided between those expressing confidence in being able to execute disaster preparedness and recovery services for events such as hurricanes even in a pandemic, and those who raised concerns about the form and function of planning for disaster preparedness and response activities during the pandemic (such as future evacuations), making it harder to plan for evacuations in the event of a hurricane. Respondents also expressed concerns about being able to see people and meet them in person as they have in the past, which is likely to be impacted by COVID social distancing guidelines, providing food and other resources to residents who shelter in place, and finding volunteers to assist in food distribution and other forms of disaster response. These responses were provided by participants with direct experience in providing disaster-related services and personally experiencing disasters, prior to the pandemic.

In assessing the impact of experience with prior disasters on responding to the pandemic, two sets of strengths and resources were identified by the study participants—those possessed by the community-based organizations (CBOs), and those possessed by the community at large. With respect to the former, several strengths of CBOs based on disaster preparedness experience and capabilities were identified. For instance, prior disaster preparedness experience and planning resources have provided a framework for how to respond and adapt to COVID, enabling some agencies to be proactive, plan ahead, and coordinate volunteers. However, some participants stated that this prior experience provided no preparation from COVID because it is so different. One of the lessons learned from prior disasters has been the need for transportable technology and the use of online platforms and social media for communication and delivery of mental health services [42,43,44,45,46]. For the overwhelming majority of agencies, however, the use of virtual platforms for telehealth or other forms of services is an entirely new experience. Some agencies have integrated COVID response into their normal disaster preparedness activities, especially with respect to dissemination of COVID-related information. For other agencies, prior disaster preparedness activities have provided a healthy donor base needed to fund new COVID-related programs. Perhaps the greatest strength that CBOs bring to bear in addressing the COVID pandemic is their engagement in partnerships with other CBOs—which may have been enhanced by the recently completed intervention trainings for the original design for C-LEARN, that for some agencies explicitly promoted partnerships across diverse types of health care and community-based agencies. Most importantly, almost all agencies reported being connected to a network of CBOs providing mental health services, food, housing, and other social services. The experience of prior disasters such as Hurricane Katrina and the Deepwater Horizon Oil Spill has demonstrated the importance of such networks in promoting individual and community resilience post-disaster [47,48], and has been identified as a key resource in responding to the COVIDp19 pandemic [49].

Several strengths and resources of the broader community served by CBOs were also identified by study participants. Past experiences with disasters and other forms of adversity have provided evidence that people can recover from disaster and made clear that skills can be acquired during disaster response and recovery, as in the instances of efforts to rebuild neighborhoods after Katrina [50]. Lei and Klopack [51] note that there is considerable evidence that prior trauma experience with natural disasters may influence the ways in which individuals respond to risks of other hazards, including the COVID-19 pandemic, by enabling them to anticipate trauma and allow them to engage coping mechanisms more effectively. Disasters and other forms of adversity can also enhance the community’s sense of self-efficacy, reflected in the willingness of residents to participate in community response efforts, such as volunteering to assist with service delivery (e.g., food distribution) [52,53,54].

However, the pandemic is also creating new challenges to preparing for and responding to natural disasters. These challenges for example include new barriers to developing evacuation plans and providing for needs of disaster victims while risking a surge in COVID-19 infections due to social distancing constraints; competing for funds to support disaster-related activities; developing new support infrastructures; and focusing on equity in disaster preparedness. Schultz and colleagues [17,55,56] have pointed to the incompatibility of simultaneously bringing people together for evacuation and sheltering during a natural disaster and keeping them apart during the pandemic. A survey of local government readiness to weather-related disasters found that small, resource-poor governments will not be able to respond well during the pandemic, leading to an increase in social inequities [49]. Thus, while CBOs have benefited from disaster preparedness training and planning and their experiences with prior disasters, the pandemic has created new and unanticipated challenges that must be addressed immediately, even in communities highly experienced in disaster response, including with community engagement strategies [23,24]. The strain on the disaster preparedness and response infrastructure caused by the pandemic could place communities vulnerable to natural disasters in particular jeopardy [57]. As noted in a recently published study of the impact of the pandemic on emergency first responders in Poland [58], addressing these challenges will require broader “out of the box” solutions that may at times deviate from standard disaster response practices and procedures.

### 4.1. Implications

As this study was conducted in the context of an ongoing community partnered research trial that aims to determine best practices and intervention approaches to build and support in disaster-prone communities in Louisiana, the findings have important implications for how that support is constructed, implemented and sustained. Several participants made note of the importance of C-LEARN trainings and webinars in helping them respond to the COVID-19 pandemic; however, there was also frequent mention of needs for additional training in addition to specific forms of support for building community resilience and meeting the pandemic-related needs of specific groups of community residents and agency clientele. Some of these recommendations could necessitate modifications to the means used by the C-LEARN collaborative or other partnerships focusing on multi-sector responses to disasters, to implement their intervention approaches in this context. Specifically, additional trainings and resources for specific needs in this pandemic or other special circumstances, may help expand best practices based on the current experience of responding to the pandemic. For instance, consideration may be given to special populations (e.g., people returning from incarceration, people living with severe mental illness) and the implementation of a stepped care approach for treatment of symptoms of anxiety relating to fears of infection and illness, the stress of isolation and confinement, and the economic consequences of the pandemic, and needs for information on best practices for safety in interaction. By adhering to CDC and FEMA guidelines for disaster preparedness and response during the pandemic [56], organizations or partnerships may be able to take on new roles supporting communities’ social distancing practices, mask use, SARS-CoV-2 testing, or immunization efforts in anticipation of threats of climate-related events such as hurricanes in 2021 and beyond. Organizations in other Gulf coast communities implemented modifications in evacuation and sheltering procedures to increase personal space and separate those with known COVID-19 infection during Hurricanes Hanna and Laura this past summer [55]. Community health workers—who are already a part of many organizations’ work in the C-LEARN collaborative and have been trained to address mental health in post-disaster contexts [59]—also might be trained in the use of universal approaches to mental health services delivery such as Psychological First Aid [60,61] or indicated approaches such as Skills for Psychological Recovery (SPR) [62] and Skills for Life Adjustment and Resilience (SOLAR) [63]—but potentially tailored to remote implementation and other information and skills features specific to the pandemic.

Specifically, the study findings have implications for natural disaster preparedness planning and execution during the pandemic. Social distancing requirements and the nationwide scale of the pandemic have both limited options for evacuating potentially exposed residents prior to the arrival of a hurricane or other extreme weather event. Community-based organizations are uncertain how to safely evacuate residents and where to evacuate them while simultaneously protecting them from risk of infection. Similarly, there is uncertainty as to how to virtually deliver certain services to survivors in the aftermath of a disaster. Development and implementation of new guidelines for disaster evacuation and support services in the context of a pandemic are warranted [49,56].

### 4.2. Limitations

The findings in this study must be interpreted within the specific context where this study took place and how the data were collected. Although numbers of participants who mentioned a particular subtheme or topic were provided, numbers alone do not necessarily reflect its salience or importance to all of the agencies represented in this study [64]. In some instances, a subtheme or topic may have been mentioned by only one study participant; yet its salience or significance must be placed in context of the larger theme in which it was clustered or categorized. Second, study participants included representatives of agencies and organizations that were members of consortium engaged in assessing the effectiveness of Community Engagement and Planning (CEP) for multi-sector coalition support, versus more standard technical assistance, to improve mental health-related quality of life among individuals at risk for depression, with exposure to social risk factors or concerns about environmental hazards in areas of southern Louisiana at risk for events such as hurricanes and storms [23]. As such, the generalizability of study findings is limited to organizations within a specific region of the United States and having some focus on community and inter-agency engagement. Further, given the rapid assessment design, we were unable to collect information on changes in needs and resources over time; what was collected reflects a specific point in time during a rapidly evolving pandemic, approximately 6–8 months into the pandemic in the United States. For instance, data were collected prior to the landfalls of a few hurricanes that have since impacted certain parts of southern Louisiana. The immediate consequences of the pandemic in responding to these impacts were not detailed in this report. However, the city of New Orleans and the surrounding region were spared the brunt of the more powerful hurricanes that made landfall in the Gulf of Mexico in the 2020 season, and many evacuees from southwest Louisiana’s brush with multiple hurricanes came to New Orleans and benefited from the resources and services identified by study participants.

## 5. Conclusions

Despite these limitations, this study has depicted the needs of both communities vulnerable to natural disasters and the agencies and organizations dedicated to preparing for and responding to such events, in terms of a supply and demand structure that has been profoundly altered by the COVID-19 pandemic. The increase in demand for disaster-related services has been accompanied by a decrease in availability of services that can be attributed to a decline in available financial resources on the one hand, and the constraints on services delivery imposed by protocols designed to prevent the spread of the SARS-CoV-2 coronavirus on the other hand. Despite the anticipated challenges to delivering services in response to a natural disaster, the networks of partnerships and prior experiences with disaster preparedness and response, along with certain features of the community that have fostered resilience to adverse events, represent key assets in coping with the pandemic and with the current hurricane season. Though limited to a particular setting with extensive experience with climate-related disasters, and preparedness and response, the lessons for interaction with a pandemic context may have important implications for approaches in other areas to consider enhancement of preparedness and response resources, trainings and partnerships in this context.

## Figures and Tables

**Table 1:** Demographic characteristics of study parishes.

Parish	2019 Population [39]	2019 Median Household Income [40]	2014 Percent of Population Living in Flood Zone [41]
East Baton Rouge	443,763	54,948	20–30
Orleans	390,845	41,604	90–100
St. Bernard	46,266	44,661	40–50
St John the Baptist	43,242	57,429	20–30

**Table 2:** Disaster preparedness and response services provided by community-based organizations in southern Louisiana.

Service	No. of Participants	Illustrative Quote
Pre-disaster		
Community education	7	“We incorporate it [disaster preparedness] into our education programming, but also just general outreach at any point during the hurricane season and right before for hurricane prep. We’ve also put together for our nutrition education, how to prepare for, or gather your supplies, healthier food options that are nonperishable. What else is there? It’s not our main focus, but we always incorporate it in” (Participant 5).
Training	5	“Our main focus is training, so we work with C-LEARN and the RADD program to do some training around resiliency and disasters” (Participant 4).
Evacuation planning and assistance	5	“One good example is Katrina. Some of our clients were being addressed at their homes. I was on one particular project called the SAIL Project via NAMI, which stood for the acronym of Supervised Adult Independent Living. And those services were targeted to persons with mental health concerns that lived in their homes or their apartments in the New Orleans Metro Area. So, one thing we did, before all staff had left New Orleans, we made sure all of those clients were either on a bus, or either with family members, or with somebody to take them out of harm’s way for Katrina” (Participant 3).
Distribution of emergency supplies	4	“Yeah, we have an emergency infant feeding kit that people can purchase. But it was a grant that they did a challenge and won and was able to get this nice booklet illustrated and create a kit that would help during disasters, primarily hurricanes for us” (Participant 18).
Assist other CBOs in preparedness planning	3	“Sure. I mean, actually I love talking about that because we partner with the Mayor’s Office of Homeland Security and Emergency Preparedness. And we have also over the past year and a half partnered with the Governor’s Office. And basically, just because we always use a nonprofit lens first, we did a very quick survey to find out which organizations actually had continuity of operations plan in place. And how prepared were they to respond to a disaster? And just in terms of looking at their own assets, and actual offices and the like. When we found out that, for the most part, nonprofits don’t have continuity of operations plans, are not ready to weather flooding event, a catastrophic flooding event or a storm. So, because of that, we teamed up with the Louisiana Association of Nonprofit Organizations, LANO, and we paid for them to update their continuity operations planning training module and offered it to nonprofit” (Participant 25).
Environmental risk management	3	“So, we tried to still work on pre disaster preparedness and mitigation. So, some of our beautification projects are environmental, we’re trying to reduce the complications of stormwater flooding. So, we’ll do bioswales, rain gardens and some DIY style projects, we can actually help filter out that water so it will not enter somebody’s home, for example or the [inaudible 00:08:47] on a small grassroots scale” (Participant 7).
Financial preparedness	3	“And then we, a few years ago, said, “We really have to dedicate some money to doing disaster preparedness.” And just working with them through scenarios, and really thinking about how to support the mayor’s office of Homeland security, and FEMA even…. And we did it in a way so that we could also, we knew that they wanted pre-positioned funding, and preregistration to get grants faster, but we used the process also to document it, and then tell our board, “Look, the request right now is that we have money in the fund, and all the information ready so that we can just press a button and make grants within 48 h for disaster.” So, we went through that profit, set it by the board. And for the last couple of emergency events, preregistered grantees got money very quickly” (Participant 25).
Post-disaster		
Distribution of food and water to survivors	5	“We’ve done a number of things and helping our clients and constituents with disaster preparedness, anywhere from providing bottled water and other products needed in case of a disaster. Whether it be a hurricane, tornado, flooding, things of that nature. We’re still doing that. We’ve also engaged with other non-profits in the community to support food distribution, and this is something we’ve done before, particularly doing tones of crisis, which could be a disaster” (Participant 1).
Debris removal and remodeling	3	“And then post disaster primarily reduce short term recovery. So, which would generically include debris removal, pulling bricks limbs, whatever that might be from the property or roadway. Gutting homes if they do get floodwater. So tearing out drywall and wet material to help that homeowners get back to a fresh start. And then we let our other partners take over for the renovation and remodeling of the flooded home. And then also with private residences, public facilities that may need help getting up on their feet. So again, schools, parks, gardeners, rec centers, Boys and Girls Clubs, other nonprofits” (Participant 7).
Mental health services	3	“I mean, there are certainly clients that have had to deal with different types of disasters, natural, man-made, and that does come up as part of the work. I don’t have a percentage, but it certainly does come up. It’s not always the referral issue, but it does become a part of the work” (Participant 26).
Rescue and recovery	3	“So, we did an initial focus on getting people safe. How do we fund efforts and coordinate those rescue operations, and so we’re really involved in helicopter evacuations and funding other efforts that would get people from their flooded homes” (Participant 16).
Health services	2	“Once we were able to get back into the city, we would do personal care type of items and distribute those. And then we moved from there to a medical mission. So, we did mobile healthcare throughout the city.” (Participant 9).
Fund raising	2	“So, after Katrina, when much of New Orleans of course was very severely impacted, and populations relocated, the Baton Rouge Area Foundation became a really central part of the response. In fact, the greater New Orleans Foundation was co-housed with us for a period of time in Baton Rouge, and we raised $44 million from people all over the country and world in order to work on rebuilding across South Louisiana” (Participant 16).
Follow up with evacuees	1	“Another thing we did during Katrina, well, we made sure that, some of our clients were in a safe place in different states. NAMI and affiliates, based in New Orleans, we made contact with the family members of the consumers to make sure they were safe. But some of them, we just could not contact for the craziness of Katrina. But some of them, we did contact and keep track of, to make sure they were doing okay. Also, for Katrina, our Westbank NAMI, the New Orleans Branch affiliate of NAMI, has two branches, one in New Orleans, Louisiana Avenue, and one in Jefferson Parish, which is Harvey, which addresses Jefferson and New Orleans communities” (Participant 7).

**Table 3:** Themes and illustrative quotes relating to impact of the COVID-19 pandemic on disaster preparedness and response.

Theme	No. of Participants	Illustrative Quote
Difficulty providing assistance virtually	8	“Okay. The only thing I’d add is that we of course struggle to be able to meet in person, and so everything has been happening virtually, which I think for the most part has not impacted the effectiveness, but it has certainly, and this is true even in organizations not responding to COVID. You see generational differences or even different levels of comfort in conducting business virtually, so that’s been a new challenge” (Participant 16).
Difficulty implementing evacuation plans	6	“What has concerned us greatly is if there is an evacuation, where do people go and not so much how they get there. I think that has been addressed in many instances with the evacuation plan, but where do they go? When you look around the surrounding states, Texas along the Gulf Coast, most of them are increasing and surging in the pandemic. And so that doesn’t seem to be a safe place to go. And travel is also another challenge. And most of the evacuation we’ve been on buses, or if people are doing their own personal evacuations, even in cars, where do you go. If you have family members, that’s probably the best shot for you to go. And there’s no guarantee. We’ve also seen that family members in the pandemic and then the whole family is quarantined or sick” (Participant 1).
Difficulty enlisting volunteers to help with response and recovery	4	“You have to have volunteers to go in and gut these houses or rebuild or whatever. Our volunteers are totally, with COVID, you can’t get them. You can’t get the corporate groups like we did. We can’t get the convention groups that we did. We would just be totally dependent on a handful of locals who could do it, and my locals are out delivering for me” (Participant 23).
Difficulty for households to acquire resources needed in the event of disaster	3	“So, our jobs with income being challenged, then people don’t have the resources to get some of the things that are needed in preparation” (Participant 1).
Has forced organizations to place disaster planning activities on hold	3	“So, if COVID-19 has affected our ability to help the programs develop their own disaster recovery plans, it has just been because there’s been so much else that needed attention now. And so, it’s not that we’ve shelved it, it’s that it’s hard to get back to it. Because every time I think, “Oh, this week I can do X,” no. This week there are going to be other crisis to address” (Participant 20).
Difficulty providing food and supplies	3	“So, for [Hurricane] Isaac, we lost power somewhere … I lost power for six days, some people had it for nine, it takes a while to get power back on, but what is that going to look like to the folks that are being fed by these food banks? And they don’t have the resources to buy food for three to five days that they’re going to need to withstand an event, like hurricanes, like I said, like a smaller scale hurricane. So again, there are a lot of issues that COVID, not directly the COVID itself, but it’s the byproduct of COVID, that’s exposed a lot of challenges within our community.” (Participant 17).
Difficulty acquiring donor support for disaster-related activities	3	“We operate primarily through partnerships and fundraising efforts. So, everything that we do is really based on fundraising, and we do that by raising funds from companies and their employees doing work-based campaigns where the employee gives a percentage of their salary to the cause of their choice. So obviously with the economic impacts that are going to come out of this, I think we’re seeing a little of that now where donors that could pledge to give are no longer giving because of their economic situations. So, I think we’ll see a trickle-down effect from our larger companies who were, in the past, giving to very specific disaster but with COVID has affected everyone. Everyone’s going to be vying for resources” (Participant 8).
Shift in funding priorities for COVID response	2	“So yeah, the answer to that is absolutely because funding’s dried up. So in order to … what’s happened is, and this is, I think why you’re going to see mission creep for a lot of organizations, is because money is very specific towards COVID and then of course local funding, or local money, has also dried up. So, you could look at foundation money, is going to very … any foundation money is primarily looking at COVID. Any kind of business money from the private sector, well they don’t have any money so what COVID has done from a non-profit perspective has been the do or die kind of thing, so now everybody’s in the COVID business” (Participant 17).
Difficulty assisting community in preparing for disasters	1	“I don’t think we’ve specifically shared anything for hurricane preparedness right now. A lot of information that we are putting out, we’re basically just using social media, has been focused around COVID relief and just trying to maintain every day now. So just making sure we’re just repeating ourselves, as everyone else is, but I think it’s still necessary to do so, but a lot of the information we’re putting out just deals with COVID and getting through COVID” (Participant 6).
Led to a greater focus on equity in disaster preparedness	1	“Other than that, I think the larger lens on this is equity. That’s where looking at disaster responses and needs of the community, how we respond to them more equitably because we know that distribution of resources is not equal across all the spaces. People are impacted in different ways. So. we’ve undergone, as an organization, putting on an equity lens to everything that we do” (Participant 8).
Disaster fatigue	1	“One of the things that is making it harder for us to plan for and respond to hurricanes this season is that people are experiencing burnout. People are getting tired of having to deal with COVID. Wearing masks has only created divisiveness in our community. Trust in institutions is faltering. It is hard to imagine coming together to respond in the event a hurricane hits New Orleans because people will need to work together.” (C-LEARN Leadership Advisory Board member).

**Table 4:** Themes and illustrative quotes relating to impact of disaster preparedness and response on response to the COVID-19 pandemic.

Theme	No. of Participants	Illustrative Quote
Resources		
Partnerships with other CBOs	12	“And the last thing I’ll say we’re part of the local VOAD, Voluntary Organizations Active in Disaster. So, we have a role to help communicate, pre- and post-disaster with fellow nonprofits that are also active from the larger level, Salvation Army, Red Cross all the way down to a smaller grassroots level having those conversations with our peers in the disaster community” (Participant 7).
Disaster training	12	“Yes, we have participated in trainings for disaster preparedness within the last year. We’ve tried to see how we can transfer some of those toolkits. Because of the pandemic. Some of them we’re able to transfer and combine the pandemic with beginning of hurricane season on June 1, we’re trying to blend those together. So, we’re also still seeing ways on how we can... Again, it’s information sharing is making people aware of the precautions they should take for both the pandemic in their health as well as hurricane preparedness. In the toolkit of hurricane preparedness, besides the usual bottled water and things of that nature, we’re also now adding mask, make sure you have mask, make sure that you have sanitizer, these types of things are put into the toolkit to make sure that you’re covering both these challenges or disaster” (Participant 1).
Mental health services	8	“Well, one of the things that spawn out after Katrina, when the city and the citizens were able to come back, we had lots of mental health concerns. So, NAMI provide that contracts with different mental health agencies, mostly the clinics in Jefferson and New Orleans Community. And we created little independent projects, services committees and services resource committees, like the SAIL Program. One of the programs to come out of Katrina was the SAIL program, which I came into after I left Houston, Texas and became a certified peer support specialist. What was different, with the SAIL program, not only do we go out to the homes of clients that have been post Katrina affected with mental health concerns, we provided housekeeping, we provided rides, transit to the grocery store, to doctor appointments” (Participant 3).
Community trust	4	“Trust. I’ve only been in this role for three years, but the organization has been around for 10 years, and my board chair, who is my mentor, and my in-person has been doing this for 30+ years. People trust her, people trust me, people trust the organization, and are willing to come to the table when you’re a competitor for the betterment of the community. And it’s because of these organizations’ response to hurricane Katrina, and Rita, and all these other disasters, that was so easily able to bring them to the table for COVID, and they feel like there is value in communicating, and I’m the only one that can do it” (Participant 24).
Response framework	2	“I think it gave us a framework for how to respond, and while, certainly you have to adapt with the COVID in mind, without that framework, we’d have to start from scratch” (Participant 16).
Healthy donor base	1	“If they’re able to respond to COVID. Probably like a healthy like donor base. And I think Katrina brought in a lot of money for habitat locally. And so, however that’s been managed over the last 15 years has got us to the point that we’re at” (Participant 2).
Lessons learned		
How to bring people together	6	“I think that the experience from Katrina of everyone coming together and working collaboratively, I mean, hundreds of residents doing this work, both created the expectation that the neighborhood association would be responsive to future disasters, but also the spirit of we come together when something like that happens. I have to say, we’ve been really, really privileged to have a lot of volunteers within the neighborhood come out to support the emergency feeding programs. I’ve had residents, week after week after week for literally months now, who are staffing the food pantry or staffing the hot meal deliveries or distributions, and that’s because I think there’s really an understanding of we look out for each other, and we have an ethos that if I’m supporting another Broadmoorian, it’s better for me too. It’s always better for all of us. So, I think we did learn a lot about how to bring people together in Katrina that we’ve been using in COVID-19, and I think that technology has also made that much more possible, had decreased some of those barriers” (Participant 18).
Response coordination	4	“There’s the Mayor’s Office of Homeland Security that runs emergency preparedness and disasters. And so, I give my support to them, but they run that, they maintain that on their own. The hospitals, because we live in south Louisiana, because of hurricane Katrina and all of the other disasters, are already thinking about and preparing that. So, the role that I play is when the disaster hits, I am a conduit to make sure that people are talking, we’re all coordinating, we’re meeting, to ensure that we’re all on the same page.” (Participant 24).
Connecting survivors to resources	4	“In other cases, we’d find out about people in the 2016 flood who’d been living in mold for months. So, connecting them to resources, getting them out of that situation and getting them healthcare is a critical type of coordination. So, all of that is to say, although COVID certainly looks different than those other disasters, we followed the same protocol, and immediately began trying to get our staff and others safe gathering together with the stakeholders and starting to put into place funding and programs that we thought would be most impactful on the ground” (Participant 17).
Increased confidence in ability to respond	4	“Well, I think all the work we’ve done prior to COVID-19, from Katrina particularly, up until this time has gotten the community to better understand that term resilience. It’s not a new term, or the definition of it is not new. People are using it more often, but people are feeling more confident that they can deal with certain challenges. And in those challenges, they’re feeling more confident that we can get through this” (Participant 1).
Information sharing	2	“I think when it comes to storm related issues or anything else, that may be coming up. We share the same information, like once we hear something that we put out what’s needed to be done in order to maintain, in order to stay safe and how you need to prepare for what’s getting ready to occur. We just share this all, right now, is just this day by day” (Participant 6).
Importance of assessing community needs	2	“I think it helped us realize that we can’t just jump in and provide what we think is necessary. Everybody after a disaster, Oh, great, let’s do such and such, but they’re not asking the people on the ground that are in need. So, it helped us to start asking questions of the people around us, what is it that you need? What could we help? Would it be useful if we did things? And before we actually jumped in and started doing them. And it was a little bit easier, I think, getting coordinated, getting people to work together” (Participant 9).
Avoid mission creep	2	“So, for us it became very obvious we have to stay in our lane and then be clearly defined on that and so again with the city and the city trying to … and I’m not saying who’s right or who’s wrong. I mean everybody’s right and everybody’s wrong in that and so it just causes some misunderstandings to happen and so in a relationship that needs to be built on trust, there was some challenges with that in the past and so for us, my lesson was stay in your damn lane and do that, do it well. Know what you do, own what you do and do it to the best of your ability and just do that. Again, you can add programs, widen your scope, no one’s against that but it’s one of these things where I don’t think people should be chasing money all the time, these one off and these organizations … I’m going to use a national organization, the American Red Cross, not the south east, I’m not talking about here, but the American Red Cross, they’re infamous for mission creep, infamous for that. Just look at what they do, you’re thinking, “How do they do this? And they do this too and they do this? What is all of this?” I’m not saying it’s right or wrong, but they do have a lot of mission creep” (Participant 17).
Reliance on technology	1	“You might say COVID 19, or I should say the whole Katrina experience of losing everything, really prepared us and put us well on the path of being a much more portable group of people down here in this region. While there’s still a technological and computer-based divide of some kind, I do find there are a lot more people these days who have laptops, who have the cellphones. You can get a message to them some kind of way, text messaging or an email or something, or any of the other newest platforms that we now have, Slack and you know” (Participant 14).
Mental health self-care	1	“Yes. Because lots of our consumers who’ve experienced Katrina, when COVID came in, they knew they had to take their medications, they knew they had to keep in contact with their resources, like case managers, social workers. And we also knew, if you’ve been through Katrina, the number one, a must thing, is to take care of your mental health, whether that is to stay inside, make sure you have groceries, make sure your medicines are stocked up from the pharmacy and make sure you have a phone. They have outer contact with somebody, to have contact, while you’re going through the COVID-19 in quarantine” (Participant 3).
Staying connected to clients	1	“And so, having had those experiences, I do think that each of the local programs have adjusted their ability to stay connected to the children they’re assigned, regardless of what else may be going on in the world. Because that was their experience with Katrina, they lost touch. They lost that connection, and so they’ve all set up systems that will allow them to maintain contact with their volunteers, and the volunteers with the children, regardless of what else is going on in the world around them. And that has helped children” (Participant 20).
How to recover in an under-resourced community	1	“So, I think South Louisiana is very unique in the diversity of disasters we’ve had. Hurricanes are predictable in New Orleans, flooding happens. We have perpetual work around those two areas. But things like a oil spill or obviously, tornadoes are hard to prepare for because they just kind of pop down and we have to respond to those, COVID. As far as the community impact of those, we know that there’s data that backs up the fact that 53% of our community are either at or below poverty or one single disaster away from slipping into poverty. Meaning that could be in blue skies, a car breaking down, a big bill falling in their lap, a big expense and a disaster we know that’s a flood at home, big expense. Interviewee: You know that’s losing your income, hurt your pocketbook and COVID. So, the trick I think with COVID is the tale of this disaster is going to be longer than we would like to have to see. With a tornado or something more tangible and physical we can sort of control response around a weather event. Controlling response around a mix of a health and economic disaster is a lot harder to predict the long-term arc of so we usually say in a disaster, there’s short term, mid-term, long term and then mitigation. Ideally, mitigation happens before any of that stuff and the cycle. And so how we recover is one variable for our community that are already highly under-resourced and then how we would mitigate going forward. So how we would prevent a similar event in the future from having a disastrous effect on our economy or our workforce, on our businesses is a whole another conversation to have but it’s a lot more complicated than building, a levee building a flood wall, clearing the storm drains and those tangible components. Interviewee: It’s really a lot of from the ground up rethinking what a healthy, equitable economy looks like for all people. Much like we’re having a conversation about what a healthy equitable police force or security would look like for our community. So, there’s a lot of those conversations which are overlapping and kind of inter playing off of each other. But we constantly think about what each disaster brings, recognizing that it affects all people, but it affects people in different ways. If you have a cushion or insurance or if you don’t because you never have those resources to even plan and prepare to begin with how that affects different people in our community differently, recognizing the entire community is affected. Because we lose our workforce, people have to evacuate and not come back, and we lose students which affects the school system and on and on and on” (Participant 7).

## Data Availability

The data presented in this study are available on request from the corresponding author. The data are not publicly available as per requirements imposed by the Louisiana State University Health Sciences Center—New Orleans that are intended to preserve participant anonymity and confidentiality.
